# Single-Access Subintimal Arterial Flossing With Antegrade-Retrograde Intervention—The Novel S-SAFARI Technique: A Case Report

**DOI:** 10.1016/j.jscai.2026.105513

**Published:** 2026-07-16

**Authors:** Shadi Halabi, Eric Gonsiorowski

**Affiliations:** a Department of Cardiology, Community Hospital, Powers Health, Munster, Indiana; b Indiana University School of Medicine, Indianapolis, Indiana

**Keywords:** case report, chronic total occlusion, critical limb-threatening ischemia, endovascular intervention, limb salvage, peripheral artery disease

## Abstract

Chronic total occlusions of tibial vessels present a major challenge in performing successful revascularization in patients with peripheral artery disease. The subintimal arterial flossing with antegrade-retrograde intervention (SAFARI) technique is an established method in which both antegrade and retrograde access are gained on either side of an occluded vessel. These 2 catheters are then “flossed” together to create a single guide wire channel across the occlusion to aid in revascularization. This report will describe a case in which the novel single-access SAFARI (S-SAFARI) technique can utilize just 1 access point via an intact pedal loop to achieve the same treatment results in patients with tibial chronic total occlusions while safely preserving their pedal loop vasculature.

## Case presentation

A 78-year-old man with a history of coronary artery disease, cerebrovascular accident, type 2 diabetes mellitus, and hyperlipidemia presented to the clinic for a nonhealing ulcer to his left first toe. His physical examination was notable for having no palpable pulse to the left dorsalis pedis artery (DPA) with the left common femoral artery (CFA), popliteal artery (POP), and posterior tibial artery (PTA) being intact. Given the risk of progression to limb loss, he was scheduled for an interventional angiographic catheterization to attempt revascularization of the diseased vessel.

Informed consent was obtained from the patient. All identifying information has been removed from images in the figure and is not discussed in the manuscript. This manuscript does not report on patients or patient data.

### Investigations

The left lower extremity arterial ultrasound showed no flow to the anterior tibial artery (ATA) and DPA with biphasic flow being noted in the left CFA, POP, and PTA.

**Figure 1:**
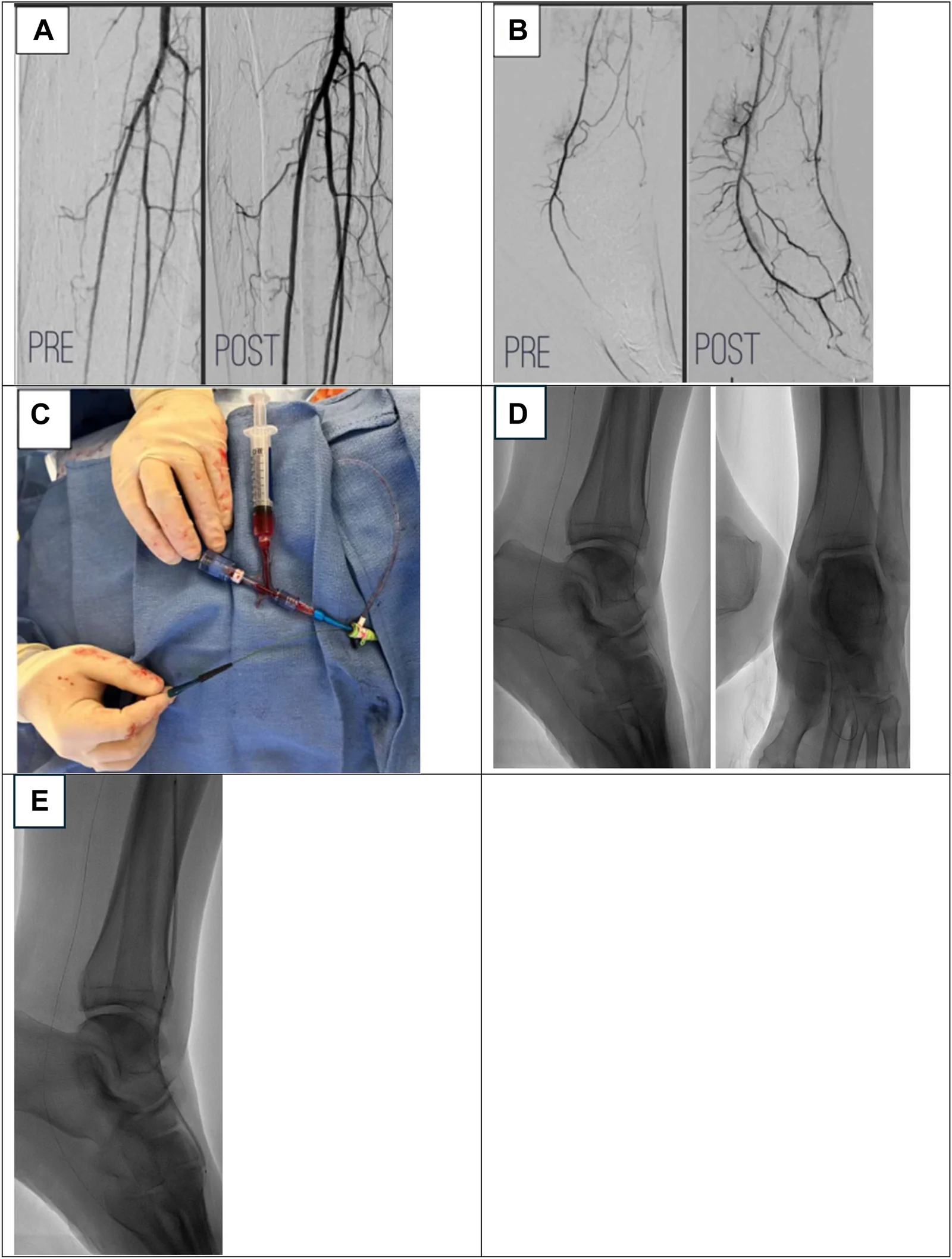
**Original images obtained during a patient case in which thesingle-accesssubintimal arterial flossing with antegrade-retrograde intervention (S-SAFARI)technique was employed.** (A) Pre- and postrevascularization of ATA. (B) Pre- and postrevascularization of DPA. (C) Two microcatheters in a 6F sheath. (D) Loop crossing confirmed in 2 orientations. (E) Balloon angioplasty of DPA and ATA. ATA, anterior tibial artery; DPA, dorsalis pedis artery.

The initial angiogram via ultrasound-guided antegrade access of the left CFA showed 100% occlusion of both the ATA and DPA (Figure 1A, B).

### Management

Antegrade attempts to cross the ATA were performed with a 0.035-inch support catheter (Navicross; Terumo) and multiple wires including the 0.014-inch (Command ES; Abbott) and 0.014-inch (Gladius Mongo; ASAHI). The interventionalist employed controlled torquing and probing of the proximal cap but was unsuccessful. Next, the single-access subintimal arterial flossing with antegrade-retrograde intervention (S-SAFARI) technique was attempted with a 0.014-inch hydrophilic wire (Sion Black; ASAHI) supported by a 0.014-inch microcatheter (CXI; Cook Medical) to advance along the patent PTA through the pedal loop into the DPA and ultimately retrogradely into the distal ATA (Figure 1C and Supplemental Video 1).

Pedal loop crossing was confirmed in 2 different projections (Figure 1D). The DPA and distal ATA occlusions were successfully crossed retrogradely with meticulous torquing of the wire. Retrograde antegrade reconstitution was achieved with the tip-in technique, in which the 0.014-inch Sion Black hydrophilic wire and support catheter were inserted into a 0.035-inch Navicross support catheter and the 0.014-inch wire was externalized (Supplemental Video 2).

Antegrade balloon angioplasty of the AT and DP was then performed, achieving brisk flow into the underperfused area (Figure 1A, B, and E). A closure device was applied to the single-access site at the superficial femoral artery. The patient was discharged on rivaroxaban, aspirin, and atorvastatin in addition to his other cardiac medications.

On follow-up, he experienced healing of the first toe ulcer with continued podiatry wound care.

## Discussion

Chronic total occlusions in the setting of chronic limb-threatening ischemia (CLTI) pose a complex challenge for peripheral endovascular interventions. CLTIs carry higher rates of limb loss and mortality.^1^ Below-the-knee chronic total occlusions (CTOs) are common in CLTI and pose a significant challenge in revascularization due to multiple factors including unfavorable caps, poor distal targets, or heavy calcifications.^2^ To address this, innovative endovascular catheterization techniques provide an opportunity to achieve successful outcomes in treating these patients. Retrograde access techniques are increasingly used as an adjunct to achieve revascularization.

The SAFARI technique, which involves antegrade and retrograde access to floss 2 microcatheters, has demonstrated success rates up to 81.9% for limb at 24 months postprocedure while even achieving improvement in the ankle brachial index, underscoring its importance in treating CTO.^3^^,^^4^^,^^5^ However, retrograde access comes with its own risks such as vessel dissection, hematoma formation, artery occlusion, and increased procedural time.^5^^,^^6^^,^^7^

The pedal plantar loop technique advocates for using the pedal loop as a conduit for crossing and advancing balloons and boasts a success rate of up to 85%.^8^ However, it requires the repeated hardware traversal through the fragile pedal plantar loop, potentially increasing the risk of spasm and dissection in these delicate vessels.^8^

The novelty of the S-SAFARI technique lies in its ability to replicate the fundamental principles of the SAFARI technique, bidirectional CTO crossing, and wire externalization while requiring only a single antegrade arterial access. In contrast to the traditional SAFARI technique, no retrograde arterial puncture is performed, and unlike pedal loop angioplasty techniques, the pedal loop is used solely as a protected internal conduit for microcatheter rendezvous rather than repeated device passage. The ability to avoid multiple arterial punctures and protection of the pedal loop add important layers of efficiency and safety to revascularization of challenging tibial CTOs.

## Conclusion

This case highlights the novel S-SAFARI technique, which enables bidirectional CTO crossing and preservation of the pedal loop vasculature through a single arterial access.
